# *In silico* design and automated learning to boost next-generation smart biomanufacturing

**DOI:** 10.1093/synbio/ysaa020

**Published:** 2020-10-17

**Authors:** Pablo Carbonell, Rosalind Le Feuvre, Eriko Takano, Nigel S Scrutton

**Affiliations:** 1 Manchester Synthetic Biology Research Centre for Fine and Speciality Chemicals (SYNBIOCHEM) and Future Biomanufacturing Research Hub, Manchester Institute of Biotechnology, The University of Manchester, Manchester M1 7DN, UK; 2 Instituto Universitario de Automática e Informática Industrial, Universitat Politècnica de València, 46022 Valencia, Spain

**Keywords:** synthetic biology, biomanufacturing, automation, machine learning

## Abstract

The increasing demand for bio-based compounds produced from waste or sustainable sources is driving biofoundries to deliver a new generation of prototyping biomanufacturing platforms. Integration and automation of the design, build, test and learn (DBTL) steps in centers like SYNBIOCHEM in Manchester and across the globe (Global Biofoundries Alliance) are helping to reduce the delivery time from initial strain screening and prototyping towards industrial production. Notably, a portfolio of producer strains for a suite of material monomers was recently developed, some approaching industrial titers, in a *tour de force* by the Manchester Centre that was achieved in less than 90 days. New *in silico* design tools are providing significant contributions to the front end of the DBTL pipelines. At the same time, the far-reaching initiatives of modern biofoundries are generating a large amount of high-dimensional data and knowledge that can be integrated through automated learning to expedite the DBTL cycle. In this Perspective, the new design tools and the role of the learning component as an enabling technology for the next generation of automated biofoundries are discussed. Future biofoundries will operate under completely automated DBTL cycles driven by *in silico* optimal experimental planning, full biomanufacturing devices connectivity, virtualization platforms and cloud-based design. The automated generation of robotic build worklists and the integration of machine-learning algorithms will collectively allow high levels of adaptability and rapid design changes toward fully automated smart biomanufacturing.

## 1. Next-generation smart biomanufacturing

**Figure 1.**
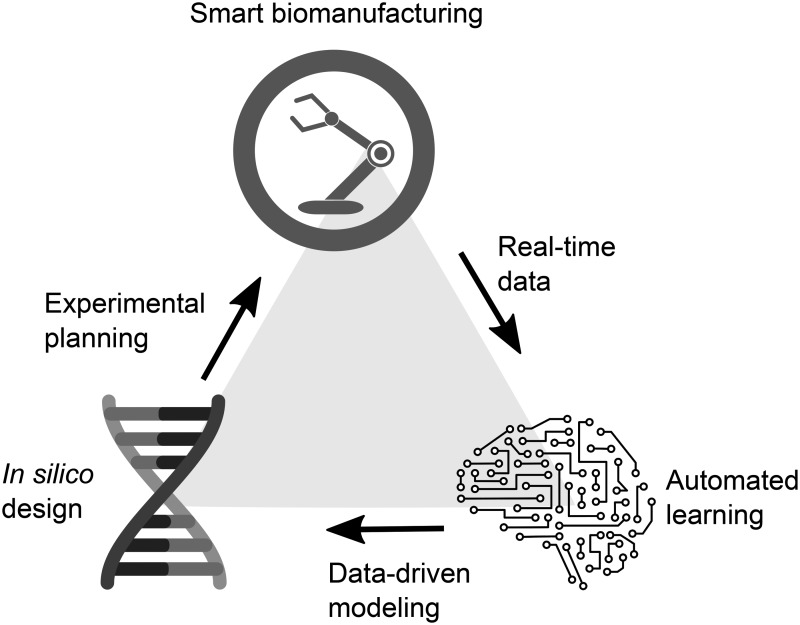
*In silico* design driven by automated learning from real-time data directs experimental planning for smart biomanufacturing.

As the global demand for bio-based products and materials steadily increases (1), a new generation of automated smart biomanufacturing laboratories is transforming the industry. Traditionally, metabolic engineering projects have been done manually, often involving trial-and-error. Today, biomanufacturing is moving to replace some of the chemical industry processes through more sustainable bio-based solutions for production not only of fine and specialty chemicals but also of commodity compounds. Biofoundries are facilities for engineering biology that aim to make such processes more efficient, systematic and standardized through the application of the Design/Build/Test/Learn cycle (2), similar to other manufacturing industries. Process automation is required in order to reach competitive time frames from initial strain screening and prototyping to scale up. Automated design for biomanufacturing is a central piece of such an approach that takes place in the initial steps of the biomanufacturing project (Figure 1). Whilst automated design has received some interest from the community (3), it is still not fully integrated due to the breadth and complexity of biological design (4), and the lack of appropriate standards for data exchange and standardized input and output formats (5). At the other end of the design, build, test and learn (DBTL) cycle, automated learning has been hindered so far because of the lack of rich datasets, i.e., big data, for training the models and the lack of interlab data in the cloud repositories allowing the gathering of information from diverse contexts (6). Here, we discuss how ongoing integration of automated design and learning will boost future biomanufacturing technologies, allowing rapid on-demand production of bespoke bio-based chemicals in smart biomanufacturing foundries.

Smart manufacturing is a technology that responds in real-time to meet changing demands (7). Biofoundries operating under the principle of smart biomanufacturing should meet the changes in the supply network for raw feedstocks as well as for synthetic genetic parts from DNA foundries; respond agilely to customers changing needs; and ensure productivity, quality, delivery and flexibility. However, meeting such demands is a challenging endeavor because of the reproducibility issues that have plagued bioproduction technologies (8) and because of the often low performance of fermentation processes in terms of titer, rates and yield and across different scales and process modes (9). Such issues are now being addressed through laboratory automation, standardization of protocols and communication between equipment. Using sensors and wireless technologies (10), cloud laboratories are being established worldwide (8) and production lines can be shared between biofoundries. Notably, the Global Biofoundries Alliance (11) is an initiative that is facilitating the collaboration between partners across the world. Several working groups are advancing towards better standardization and interoperation of software for controlling production processes and resource planning.

Automated optimization of the processes is becoming possible through robotic fermentation platforms, where growing conditions can be highly monitored and controlled in a parallelized fashion with early consideration of scale up (12). The wealth of big data that current omics techniques provide allow the real-time analysis of proteomics, transcriptomics and metabolomics data. However, high-throughput screening can be a limitation for certain classes of molecules, for instance, if no biosensor exists. Powerful machine-learning algorithms in combination with automation can then be used to exploit the data and predict new interventions for process optimization (13). In recent years, the number of applications of machine learning into biotechnological processes has notably increased thanks to a new generation of algorithms such as deep learning as well as the deployment of easy-to-use development frameworks (14). Deep learning algorithms are a family of neural networks methods where layers are generalized and organized in complex architectures such as convolutional networks. For instance, deep learning was applied by Wu *et al.* (15) to assist the directed evolution of an enzyme in order to produce the two enantiomers of a new-to-nature carbene Si-H insertion reaction. Sampling the combinatorial sequence space by directed evolution alone would have been too expensive, but machine-learning models trained on tested variants provided a faster method by allowing its computational exploration (16). Similarly, deep learning has been applied to biological sequence data (10) in order to perform functional prediction. Amidi *et al.* (17) used 3D convolutional networks, a class of deep learning algorithms, to classify enzymes by their EC class in the entire PDB database based on spatial structure. Machine learning has been applied to different strategies for metabolic engineering (18), for instance, Costello and Martin (19) combined machine learning with multiomics data (proteomics and metabolomics) to predict the kinetic model of engineered metabolic pathways. In yet another example, Opgenorth *et al.* (20) used the data produced in the first Design/Build/Test/Learn cycle to train several machine-learning algorithms and to suggest protein profiles for a second cycle that would increase production. Similarly, the cell-free iPROBE platform for prototyping and rapid optimization (21) implemented a neural network to predict beneficial combinations of biosynthetic enzymes in a pathway. Several examples also exist of the application of machine learning to synthetic biology, such as in the prediction of success of rapid synthesis of long DNA fragments (22) the lab-of-origin of the engineered DNA (12), or in the design of simultaneously stable single-guide RNAs for CRISPR interference (23), context-aware DNA design in translational regulation for limonene production in Escherichia *coli* (24), as well as in other strategies for the design of gene circuits (25).

## *2. In silico* design tools for biomanufacturing

As a major contributor to the new fully automated pipeline paradigm, the concerted orchestration of a growing ecosystem of *in silico* design tools is undeniably transforming metabolic engineering into a biomanufacturing technology. For some years design tools were considered as simply assistants for individual tasks, but the current view is that integrated *in silico* tools should underpin the automated pipeline for the design stage of smart biomanufacturing. The first task of metabolic pathway design automation is the selection of chemical targets. Generally, targets are imposed by external partners such as customers interested in commercializing the bio-based version of some compound of interest like a natural product, a pharmaceutical or a material building block, to name a few. Another consideration in the development of sustainable industrial systems is the use of sustainable feedstocks or utilization of waste products in a bid to reduce resource consumption (including reliance on precious metals and decreasing petrochemical consumption) and greenhouse gas emissions, this is increasingly important in the bid to mitigate climate change and ensure clean growth (26). Recently, several proposals have appeared trying to rationalize automating the target selection in the same fashion as in the chemical industry. Life cycle and techno-economic analyses (27) are critical steps used to assess the potential economic viability of production by microbial fermentation of the target from raw materials at an industrial plant scale, as well as assessing the value of new chemical production routes that circumvent existing intellectual property (28). Whilst these are at present mostly performed manually they show potential for achieving automated target selection (29).

Moreover, the bio-based production of chemical targets requires the identification of viable routes. It is possible that a natural biosynthetic pathway exists, but it might have been optimized through evolution in a different host (30). Therefore, even when routes are available, it is often preferable to select enzymes from different sources. A more ambitious effort consists of looking for alternative pathways other than the natural one or the engineered ones reported in the literature. To that end, retrosynthetic analysis is performed (31). Several computational platforms for retrobiosynthesis exist (32, 33), such as BNICE (33), GEM-Path (34), RetroPath 2.0 (35), PathPred (36) or Cho *et al*. (37). Each one differs from the others on their unique computational representation of reactions and the way predicted pathways are ranked. However, there is a sizable gap between the virtually huge number of alternative routes that can be computationally predicted and the few that because of limitations in the total number of experimental runs to be performed would finally be tested in the laboratory. Because of this challenge, retrosynthetic algorithms have been increasingly refined with the hope of becoming ‘smarter’ and therefore capable of providing routes that are the closest to the expert suggestions. Using state-of-the-art machine-learning algorithms such as deep learning, it has been shown recently that automated retrosynthetic analysis for synthetic chemistry can beat the experts (38). Retrosynthetic analysis for biosynthesis, i.e. bioretrosynthesis, is more challenging because it needs to combine chemical reaction knowledge with enzyme biocatalysis expertise. Despite this complexity, recent progress using reaction rules with associated scores is helping in narrowing down the search to the most promising routes (39), as exemplified in RetroPath2.0 (35).

When performing pathway selection, it would be of little use to select one of the predicted pathways through retrosynthesis if enzymes with the required efficiency cannot be found for individual steps of the pathway. Enzyme selection can be done based on homology, i.e. by performing a BLAST search of homologs, as performed by EC-Blast (40) and then selecting them either because of their reported activity in databases like BRENDA or any diversification approach. Moreover, the search can be complemented by looking for reactions that are similar to the target reaction. This is the strategy developed by Selenzyme (41), which is well-fitted for annotating retrosynthetic searches that are based on reaction rules. This tool provides key information about enzymes based on several criteria such as a reaction and sequence similarity; phylogenetic distance between source organism and host strain; sequence conservation; and other relevant properties. Other sources of information for the enzyme selection include structural analysis of the ensemble of homologs and prediction and identification of active sites and beneficial mutations. Enzymes can be screened for efficiency, and directed evolution (42) is generally then used in order to evolve variants with improved efficiency with respect to the native counterpart by defining the set of amino acid regions in the enzyme sequence to be mutated in order to improve its functionality. Such a process can be automated by using technologies like deep learning (15, 43). Several methods based on deep learning have been proposed for enzyme sequence design, for instance, to classify enzymes by EC number based on sequence (44, 45) or in enzyme engineering (46).

*In silico* enzyme selection is generally followed by genetic parts selection, both at transcription and translation levels. Transcriptional selection typically involves promoter design (47) using tools such as SelProm (48) or ePathOptimize (49), which has been applied to the violacein pathway. Translation design tools like the RBS Calculator (50) and RBSDesigner (51) are facilitating the bottom-up genetic circuit design. Tools are also guiding the design of smart combinatorial genetic circuit libraries like RedLibs (52) and PartsGenie (53) as well as automated circuit design such as include Cello (3), which can automatically compute the circuit implementing the desired logic function. All of these tools combined together with the increasingly more efficient high-throughput capabilities of smart biomanufacturing platforms, are nowadays optimized by the design of experiments (DoE) approaches (54). Through the optimal experimental design, smart biomanufacturing platforms can rapidly develop high producer titers based on a reduced set of experimental runs. The optimal design will generally follow the strategy of assuming simple models and relationships between the factors for rapid prototyping and identification of main factors among gene variants, promoters and other genetic parts. Those main identified factors can be then explored in depth through focused combinatorial libraries in order to model complex interactions through machine-learning algorithms.

## 3. The future of biomanufacturing is smart

Microbial strain engineering for on-demand production of chemicals is a major focus of biomanufacturing foundries that are developing automated pipelines that are agnostic to the target compound (2). Ultimately, the suite of fully automated design protocols would be integrated in the pipelines to allow seamless transition from chemical target to automated generation of build workflows. In addition, the development of cloud-based platforms would allow the remote implementation of design stages that feed into distributed (or distant) wet-lab facilities with experimental data automatically feeding back into learning steps for optimized re-design and iterative cycles for optimization (8, 55). Broadening the portfolio of design tools and providing access will allow the sharing of capabilities and the development of virtual *in silico* design. However, the wide uptake of design platforms will depend on the standardization of biological engineering and ensuring that these tools are accessible to the non-expert user.

**Figure 2.**
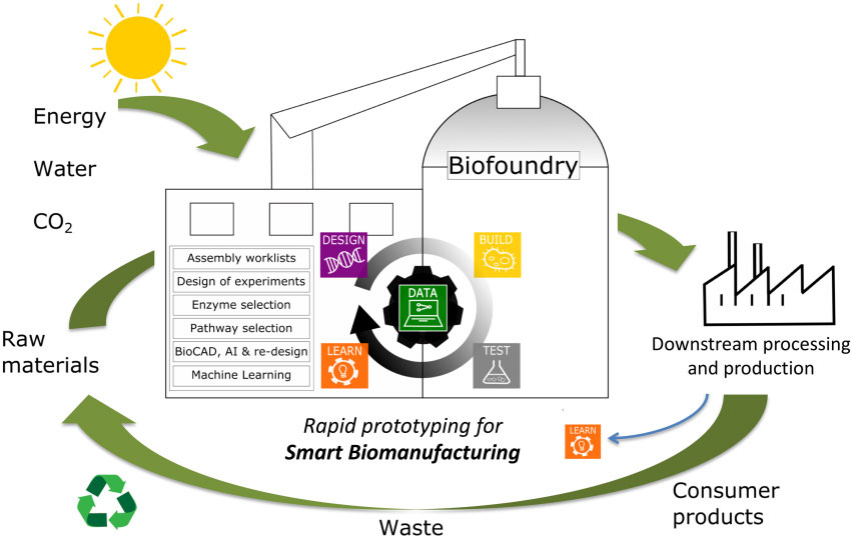
Biofoundry model for rapid prototyping and smart biomanufacturing based on the automated Design, Build, Test and Learn (DBTL) cycle integrated with high-dimensional data and the principles of the circular economy. A suite of *in silico* machine learning-driven BioCAD tools for parts selection, build assembly and experimental test can leverage the agile circular process for downstream processing (DSP) and production of consumer products in order to achieve sustainable solutions that utilize renewable/waste feedstocks and replace the use of fossil fuels.

Towards that end, several initiatives have been proposed in order to define metrics and assess the capabilities of biofoundries. In one of such initiative, a timed ‘pressure test’ administered by the US Department of Advanced Research Projects Agency (DARPA) consisting on building organisms to produce 10 molecules ran for 3 months in order to assess the foundry (56). Interestingly, several approaches were applied including retrosynthetic design and cell-free systems, achieving an outcome of 6/10 targets produced during the performance period. As a recent milestone, the Manchester SYNBIOCHEM Centre focused its DBTL pipeline on the rapid prototyping of microbial factories for the biosynthetic production of monomers for enhanced biomanufacturing of materials (57). The *in silico* design phase from initial target and pathway selection through to the design using tools such as RetroPath2.0, Selenzyme and PartsGenie and ordering of DNA parts took ∼10 days. Over 85 days, more than 160 genetic parts were designed and assembled into 115 unique biosynthetic pathway constructs and tested for *in vivo* production in *E. coli.* Targeting chemically diverse industrially relevant materials building blocks, the Centre’s rapid prototyping capability successfully produced 17 materials monomers at competitive titers and key intermediates to demonstrate the potential of biofoundries in leading the sustainable production of next-generation materials. The ability to produce strains for gateway chemicals (e.g. flavanones (58)) with broad applications such as precursors to materials and active pharmaceutical ingredients, will be a major step forward. One such materials monomer targeted was enantioselective production of mandelic acid, a monomer for degradable thermoplastics with polystyrene-like properties (59) that can also serve in its enantiopure form as a building block for the synthesis of pharmaceuticals (60), with rapid scale-up delivery of g/l quantities in bioreactor cultures achieved. Whilst the potential for biobased production of key compounds has been demonstrated, in order for bio-based manufacturing to become a reality, the whole ecosystem of production needs to be considered with substantial reductions in the costs of production to become commercially viable (Figure 2). Important considerations include both upstream and downstream processes including the development of new more environmental friendly solvent/extractant systems with reduced energy consumption and loss (61, 62); greener product recovery (63) and separation processes (64); choice and cost of feedstocks and substrates; and the potential to utilize low/zero-cost waste streams (e.g. lignocellulose and glycerol waste from biodiesel production), or CO_2_ (65–67). The engineering of robust industrial microbial strains for chemicals production at scale and to reduce the economic burden of biomanufacturing will require systems level design and implementation of integrated bioproduction platforms (56). Full techno-economic assessment will also be important to consider the commercial viability including production modes, separation technology and the price point of target molecules (e.g. commodity vs high value). Standardization, robotics and automation which will play an important part (8) in the development of ‘smart biomanufacturing’, are the focus of many research groups around the world, including the Global Biofoundry Alliance (https://biofoundries.org). Finally, coupling to ‘greener’ downstream processing (e.g. (68)) and waste recycling will be essential for the successful delivery of a circular biobased economy.
